# DNA methylation-estimated phenotypes, telomere length and risk of ischemic stroke: epigenetic age acceleration of screening and a Mendelian randomization study

**DOI:** 10.18632/aging.206072

**Published:** 2024-08-16

**Authors:** Aierpati Maimaiti, Jianhua Ma, Chenguang Hao, Dengfeng Han, Yongxin Wang, Zengliang Wang, Rena Abudusalamu

**Affiliations:** 1 Department of Neurosurgery, Neurosurgery Centre, The First Affiliated Hospital of Xinjiang Medical University, Urumqi, Xinjiang 830054, China; 2 Department of Neurology, The First Affiliated Hospital of Xinjiang Medical University, Urumqi, Xinjiang 830054, China

**Keywords:** epigenetic age acceleration, ischemic stroke, methylation clock, mendelian randomization, stroke subtypes

## Abstract

Background: Aging is a complex biological process that may be accelerated in certain pathological conditions. DNA methylation age (DNAmAge) has emerged as a biomarker for biological age, which can differ from chronological age. This research peels back the layers of the relationship between fast-forward aging and ischemic stroke, poking and prodding the potential two-way causal influences between stroke and biological aging indicators.

Methods: We analyzed a cohort of ischemic stroke patients, comparing DNAmAge with chronological age to measure age acceleration. We assessed variations in age acceleration among stroke subtypes and between sexes. Using Mendelian randomization, we examined the causal links between stroke, aging biomarkers like telomere length, and age acceleration's effect on stroke risk.

Results: Our investigation reveals a pronounced association between ischemic stroke and age acceleration, most notably in patients with cardioembolic strokes, who exhibited a striking median difference of 9 years between DNAmAge and chronological age. Furthermore, age acceleration differed significantly across stroke subtypes and was higher in women than in men. In terms of causality, MR analysis indicated a modest negative effect of stroke on telomere length, but no causal effect of age phenotypes on stroke or its subtypes. However, some indication of a potential causal effect of ischemic stroke on PhenoAge acceleration was observed.

Conclusion: The study provides insight into the relationship between DNAmAge and ischemic stroke, particularly cardioembolic stroke, and suggests possible gender differences. These insights carry profound clinical significance and set stage for future investigations into the entwined pathways of stroke and accelerated aging.

## INTRODUCTION

Ischemic stroke (IS) represents the primary cause of disability and mortality on a global scale [1], accounting for 10% of disability-adjusted life-years lost and 5% of deaths each year [2]. The identification of fundamental risk factors and protective elements is crucial in developing effective prevention strategies for stroke, given its increasing global impact. This is emphasized in literature [3]. The preeminent risk factor for ischemic stroke is chronological age, although the mechanisms responsible for this association are not yet fully understood [4]. Gaining a more profound comprehension of this variability may provide significant perspectives into the causes of stroke, thereby enabling the creation of remedial measures that can alter and potentially avert age-related health hazards [5].

Epigenetic clocks utilize mathematical models that incorporate the DNA methylation (DNAm) state of Cytosine-phosphor-guanine (CpG) dinucleotides to forecast certain aspects of chronological age and clinical phenotype. Certain CpG sites exhibit a significant association with actual age [6]. Although biological age, as determined by DNA structures at specific CpG sites, may differ from chronological age, empirical data suggests a correlation between accelerated epigenetic modification aging (i.e., when biological age surpasses chronological age) and heightened susceptibility to mortality and age-related ailments [7]. Epigenetic clocks are utilized as inheritable markers of biological aging that rely on DNAm data. These clocks are generated through assessments of DNAm levels at particular CpG loci that encompass unique epigenetic aging characteristics [8]. The initial versions of genetic chronometers, such as HannumAge [9] and Intrinsic HorvathAge [10], were developed by utilizing DNAm levels obtained from various groups of age-associated CpG loci that demonstrate a robust association with chronological age. The HannumAge model was formulated utilizing 71 age-associated CpGs that were detected in blood [9]. On the other hand, the Intrinsic HorvathAge model was constructed using 353 CpGs that were found to be related to age across various body tissues and cell types. Furthermore, this model was adjusted to account for blood cell counts [10]. During the period of 2018-2019, novel genomic detectors were developed, namely PhenoAge [11] and GrimAge [12], which belong to the second generation of such detectors. These detectors were designed to forecast the likelihood of mortality and morbidity that are linked to the process of ageing. The PhenoAge dataset comprises information collected from 513 CpG and 9 clinical parameters, including albumin, alkaline phosphatase, C-reactive protein, creatinine level, hemoglobin transportation dimension, mean hemoglobin quantity, bloodstream glucose, lymphoid proportion, and white blood cell count, which are associated with mortality [11]. On the other hand, GrimAge dataset incorporates data from 1,030 CpG and 7 plasma proteins that are linked to smoking. The proteins in question are cystatin C, β-2-microglobulin, tissue-associated protease 1, adrenomedullin, development division protein 15, and fibrin stimulation inhibitor 1 (PAI-1) [12]. The evaluation of accelerated ageing encompassed the development of DunedinPACE, a third-generation biomarker for DNA methylation that calculates the pace of ageing through epigenomic means. The Dunedin Longitudinal Study Cohort was utilized to measure 19 markers across multiple bodily systems (including cardiovascular, metabolism, liver, kidneys, immune, periodontal, and pulmonary) in a continuous manner among individuals aged 26, 32, 38, and 45 years old. This information was documented in a previous study [13]. The information yielded a model for ageing rate that is based on a specific DNA methylation biomarker, which captures age-related changes across various biological systems.

HannumAge and Intrinsic HorvathAge exhibit superior predictive capabilities for actual age owing to their distinct composition, while PhenoAge and GrimAge demonstrate exceptional performance in forecasting health and longevity, as reported in literature [14]. The acceleration of HannumAge, Intrinsic HorvathAge, PhenoAge, and GrimAge has been found to be associated with age-related illnesses, as per previous research [7]. The results suggest a potential positive correlation between DNAm Age and age-related disease prevalence, although the findings exhibit some variability. Comparisons that are specific to diseases pose a challenge due to the significant heterogeneity of outcomes, even within disease categories. Two studies pertaining to ischemic stroke were conducted with differing objectives. One study aimed to determine the incidence of ischemic stroke [15], while the other focused on assessing the severity of ischemic stroke outcomes during follow-up [16]. Despite variations in study populations and outcomes, with the exception of one study, all studies arrived at the conclusion that an increase in DNAm Age is indicative of heightened risk for future illnesses. These findings are consistent with mortality and offer further evidence for the potential of DNAm Age as a global biomarker for biological aging and health.

Furthermore, telomeres, as repetitive sequences at the ends of chromosomes, gradually shorten with cell division. Telomere length is considered a marker of cellular aging and regenerative capacity. Studies have found that telomere shortening is closely associated with the occurrence of various chronic diseases, including cardiovascular diseases and ischemic stroke [17, 18]. Telomere shortening may lead to cellular dysfunction and inflammatory responses, which are important mechanisms in the occurrence of stroke [19].

Integrating studies on DNA methylation and telomere length can provide a comprehensive perspective, revealing their joint mechanisms in the occurrence of ischemic stroke. Changes in DNA methylation may affect telomere length by regulating gene expression, thereby influencing cellular aging and function. Additionally, environmental and lifestyle factors may jointly influence stroke risk by affecting DNA methylation status and telomere length [20, 21]. By studying these two biomarkers, we can better understand the pathological mechanisms of ischemic stroke and develop more effective predictive and preventive strategies.

The strength of the association between accelerated methylation age and age-related diseases is found to vary depending on the methylation clock, as per recent findings. The correlation between epigenetic age velocity and age-related ailments, such as tumors, appears to be notably stronger when determining biological age using second-generation clocks (PhenoAge and GrimAge) as opposed to first-generation clocks (HannumAge and Intrinsic HorvathAge) [22]. The lack of agreement among genetic clocks could be attributed to the diversity in their research designs and the differences in the techniques employed, which may also indicate distinct physiological senescence mechanisms. This heterogeneity may contribute to the variability observed in the results [7]. Despite the consensus among researchers, the causal relationship between DNA methylation and age-related disease risk remains uncertain. It is yet to be determined whether DNA methylation serves as a predictive biomarker without any causal effect.

Mendelian randomization is an epidemiological technique that employs genetic variations as instruments to establish correlation between a risk factor (exposure) and an outcome of interest, subject to specific assumptions. This technique holds significant value in the field [23]. The MR method relies on three basic assumptions: (I) the instrumental variables (i.e., SNPs) are closely related to the exposure factors (e.g., DNA methylation); (II) the instrumental variables affect the outcome only through their influence on the exposure factors; (III) the instrumental variables are independent of confounding factors [24]. However, measurement biases and differences in data formats across platforms need to be corrected through standardization and normalization processes. To this end, we utilized various bioinformatics tools and software packages, such as EasyQC and METAL, to ensure data consistency and accuracy [25]. To ensure that the instrumental variables influence the outcome only through the exposure factors, we conducted sensitivity analyses, such as MR-Egger regression and weighted median estimation, to assess potential pleiotropy (i.e., the instrumental variables affecting the outcome through other pathways) [26].

The findings of a meta-analysis conducted on genome-wide association studies (GWAS) indicate that there are 137 genetic regions that are associated with methylation age acceleration. These regions were estimated using six regulation indicators and can be utilized in the MR framework [27]. The current study investigated the causal relationship between epigenetic age acceleration and stroke, including ischemic stroke subtypes such as cardioembolic stroke, large-artery atherosclerosis stroke, and small-vessel disease stroke, through a two-sample bidirectional Mendelian randomization analysis. In addition, our study examined the causal impact of stroke and its various subtypes on diverse indicators of epigenetic age acceleration. The study’s results offer new perspectives on the intricate connection between the process of biological aging and the likelihood of experiencing a stroke. These insights may have significant implications for developing strategies aimed at preventing and treating strokes.

## METHODS

### Data collection and *in silico* DNA methylation analysis

The methylation status of 353 CpG sites related to age was analyzed *in silico* through the utilization of Illumina BeadChip450K methylation array data to determine the DNA methylation condition [10]. The methylation levels of these sites are significantly correlated with age. The selection of these sites is based on their consistency and stability across different tissues and cell types, ensuring the model’s applicability and accuracy across different individuals [9, 10]. Existing research indicates that many age-related CpG sites are not only markers of biological age but are also associated with various age-related diseases, such as cardiovascular diseases and neurodegenerative diseases [28, 29].

A cohort of 185 patients diagnosed with ischemic stroke underwent whole-genome methylation sequencing of whole blood using the Illumina HumanMethylation450 BeadChip (HumanMethylation450_15017482), from which data was collected. The NCBI Gene Expression Omnibus Database (GEO accession number: GSE69138 [30–32]) revealed that among the patients with available samples, there were 109 cases of atherothrombotic stroke, 18 cases of cardioembolic stroke, and 58 cases of small-vessel stroke. The demographic and clinical characteristics of each patient were documented, encompassing factors such as age, gender, and the subtype of stroke.

### Methylation clock analysis

We employed Horvath’s methylation clock model to calculate the DNA methylation age (DNAm Age) for each patient, an estimation of their physiological age. We determined age acceleration by comparing DNAm Age with chronological age, using two distinct measures: Relevant calculation formula: Age Acceleration Diff = DNAm Age - Chronological Age.

Age Acceleration Residual = residuals (lm(as.numeric (df$DNAm Age)~as.numeric(df$Age), subset= df)). Age Acceleration Diff, calculated as DNAm Age minus chronological age, and Age Acceleration Residual, derived from the residuals of a linear regression model (using the lm function in R) with DNAm Age as the dependent variable and chronological age as the independent variable. Age Acceleration Diff directly reflects the disparity between DNA methylation age and chronological age. If the Age Acceleration Diff is positive, it indicates that the biological age is greater than the chronological age, which may imply an accelerated aging process; conversely, a negative value suggests a slower aging process. In statistical analyses, this metric helps identify individuals with accelerated biological aging and explore its association with stroke risk [10, 11]. Age Acceleration Residual, after adjusting for the effect of age on DNA methylation age, more accurately reflects the degree to which an individual’s biological age deviates from its expected value. In statistical analyses, Age Acceleration Residual is used to assess the potential relationship between abnormal acceleration of biological age and stroke risk, excluding the confounding effect of chronological age [20]. The F test was utilized to figure out the correlation that exists between locus-by-locus variation in DNA methylation and stroke longevity or age of beginning, as well as to assess the erroneous discovery rate in order to produce an adjusted *q*-value that corrects for multiple comparisons. The *F*-test can effectively detect significant variance differences between groups, thereby helping to identify potentially important DNA methylation sites [33]. The coefficient of correlation of Pearson was applied to estimate the causal connection among DNAm age-acceleration and stroke longevity or age of onset. Using linear regression, it was determined whether the correlation matches a linear model. For each CpG site (cg IDs), differences between the mean mutation estimates of the contrasted diagnosis groups (Δβ-values) and the values of *P* were determined. For statistical evaluation, the Kolmogorov-Smirnov test was utilized to confirm normal distribution. In all instances where a normal distribution was observed, a Student’s *t*-test in Benjamini and Hochberg adjustment was used to compare paired groups. In all cases, significance criteria were *P* < 0.05. Additionally, we validated the reliability of the methylation clock in aging outcomes by collaborating with Andrew E. Teschendorff [34] and Jamaji C. Nwanaji-Enwerem [35], using EpiTOC2 to measure DNAm changes related to mitotic activity.

### Mendelian randomization study design

To further explore their causal relationship, we performed two-sample bidirectional MR of this study. In short, we utilized GWAS data of DNA methylation to explore the causal relationship between age-related phenotype and stroke and its subtypes.

### Data sources for epigenetic age acceleration

The present study obtained condensed genetic correlation estimates for intrinsic environmental age acceleration, specifically HannumAge, PhenoAge, and GrimAge, from a recent meta-analysis of GWAS pertaining to biological aging. The meta-analysis encompassed a sample of 34,710 individuals of European descent and 6,195 individuals of African American descent [27]. Furthermore, the selection of DNAm PAI1 levels was based on its stronger correlation with cardiovascular and metabolic disorders when compared to epigenetic modification clocks [12]. Additionally, granulation cells proportion was chosen due to its significant interactions across several epigenetic timers, including GrimAge and PhenoAge [11, 12]. The primary source offers a comprehensive account of the methodologies employed. Age-adjusted DNAm estimates were computed using the Horvath genetic age estimator program (https://dnamage.genetics.ucla.edu/) or autonomous scripts. Individuals whose circadian methylation estimates deviated by more than 5 standard deviations from the mean were excluded from subsequent analysis. Single nucleotide polymorphisms (SNPs) have been identified and differentially calculated for each cohort in the conducted meta-analysis. The genotypes in all cohorts, except for the Sister Study and the Genetics of Lipid Lowering Drugs and Diet Network Study, were imputed using either the HRC or 1000 Genomes Project Phase 3 reference panels. The Sister Study lacked estimated data at the time of the study, while the Genetics of Lipid Lowering Drugs and Diet Network Study utilized whole-genome sequencing data. GWAS summary statistics were obtained from each population through the utilization of multiplicative linear regression models that took into consideration sex and genomic principal components. The statistics were processed and standardized using the ‘EasyQC’ R package, followed by conducting ascertained-effect systematic reviews using the METAL program [25]. Moreover, short telomere length is a well-established trigger of replicative senescence [36], we further obtained genetic association estimates for epigenetic telomere length from UK Biobank which consisted of 472,174 participants. We followed the method outlined by Sudlow, C., et al. for the measurement and processing standards of epigenetic telomere length in the UK Biobank (UKB) data [25, 37, 38].

### Data sources for stroke

We derived stroke, ischemic stroke and common etiological subtypes of ischemic stroke include large-artery atherosclerotic stroke (LAS), cardioembolic stroke (CES), and stroke caused by small-vessel disease (SVS) from a multiancestry genome-wide-association meta-analysis in 521,612 individuals (67,162 cases and 454,450 controls) [39]. The original publication expounded upon the comprehensive analytical approach. The studies included in the analysis employed genotypes that were attributed to no less than the 1000G phase 1 multiancestral reference panel. Statistical analyses were conducted using logistic regression (or Cox regression analyses for long-term population-based cohort studies) for five stroke characteristics. All determined and imputed variations in genes were used in dosage format, and appropriate programs were utilized under a combined genetic model with at least sex and age as covariates.

### Genetic instruments

In order to assess the linkage disequilibrium (LD) among SNPs, the researcher identified SNPs that exhibited a significant association with stressors at the genetic sequence-wide significance level (*P* < 5 × 10−8). Subsequently, a clumping process was performed (R^2^ < 0.001, window size = 10000kb) utilizing data from the European population that was collected for the 1000 genomes project. Using the PLINK command, identify SNPs within the set window that have an R^2^ greater than the threshold with the seed SNP, and retain only the most significant SNP. The purpose of this step is to identify SNPs within the set window with an R² greater than the threshold using the PLINK command and retain only the most significant SNPs. This method helps reduce issues related to multiple comparisons, simplifies the analysis process, and enhances statistical power [40–42]. In instances where the instrumental SNP associated with the degree of exposure was not detected in the resulting dataset, we either replaced it with a suitable proxy SNP (r^2^ > 0.8) or removed it altogether. In order to assess sensitivity and identify any potential breaches of the presumption of relevance, we conducted an analysis by calculating F-statistics (F= (β/se)^2^) for all measurements of genetic generation progression, with the exception of the experimental variable (F < 10), which was deemed to be weak. The SNP genotypes were standardized across studies and palindromic SNPs with unclear allele frequencies were eliminated.

### Statistical analysis

The study employed exponentially random impact inverted variation weighted (IVW) Mendelian randomization (MR) to investigate the association between accelerated epigenetic age and stroke risk across genetic variants [43]. Several alternative methods for MR have been proposed to address the issue of directional pleiotropy. These methods include MR-Egger, Weighted median, Weighted mode, and Simple mode. The Cochran Q test was employed in the context of the instrumental variable analysis to ascertain the presence or absence of horizontal pleiotropy. The study employed the MR-Egger, MR-Pleiotropy Residual Sum, and Outlier methods to examine horizontal pleiotropy [44, 45]. A significant Egger intercept indicates the presence of directional and unbalanced horizontal pleiotropy. The calculation of the effect estimate involves the utilization of MR-Pleiotropy Residual Sum and Outlier methodology, which entails the identification and subsequent exclusion of outlier SNPs, also known as putative pleiotropic variations. The study employed a “leave-one-out” sensitivity analysis to identify potentially significant SNPs by iteratively conducting MR while excluding each SNP. The R^2^ values were computed by augmenting the EAF values with 2 × EAF × (1 - EAF) × β^2^. The statistical software R was utilized to execute the studies, employing the programs “TwoSampleMR,” “Mendelian randomization,” and “MRPRESSO.” Statistical significance was determined by *P*-values < 0.05. We used G × Power software to calculate the statistical power with an expected medium effect size (Cohen’s d = 0.5), significance level (α = 0.05), ensuring power exceeding 80% [46]. Based on existing studies associating biological aging and stroke risk, along with data from 34,710 samples of European descent and 6,195 samples of African American descent, we conducted sample size calculations to ensure detection of associations of medium effect size [47]. To address the complexity of the relationship between epigenetics and disease, we employed multiple testing correction methods (such as Benjamini-Hochberg correction) to control the false positive rate due to multiple comparisons [48].

### Data availability statement

The article/Supplementary material contains the original contributions made to the study. Corresponding authors can be contacted for more information.

## RESULTS

### Age acceleration in ischemic stroke patients

**Figure 1:**
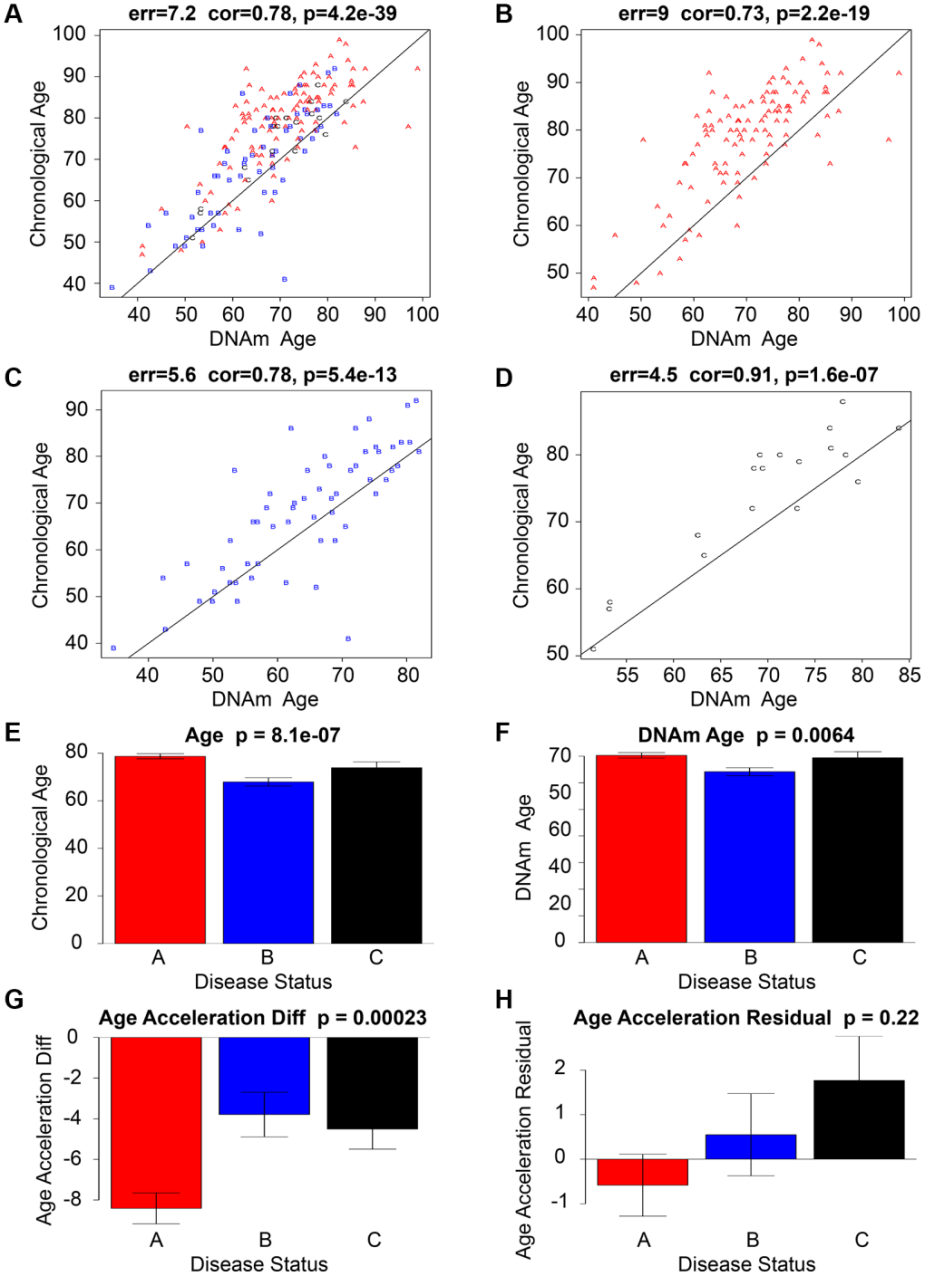
**An analysis of a data set pertaining to ischemic stroke.** The scatter graphs presented in the top row (**A**–**D**) of the Ischemic stroke data sets depict the relationship between DNAm age (x-axis) and chronological age (y-axis). A represents a cardioembolic stroke and is colored red, B represents a small-vessel stroke and is colored blue, while C represents an atherothrombotic stroke and is colored black. The line of regression through IS is represented by the black line. The vertical distance to the black regression line corresponds to the effect of age acceleration for each subject. Although there is a strong correlation between chronological age and DNAm age, it has been observed that Red, Blue, and Black Alphabet tend to exhibit accelerated aging effects as they lie above the black line. The lowermost tier (**E**–**H**) depicts the correlation among chronological age, DNAm age, Age Acceleration Diff, Age Acceleration Residual, and the presence or absence of Ischemic Stroke Disease. The bar graphs’ titles comprise the *P*-value obtained from a nonparametric group comparison test, specifically the Kruskal-Wallis test.

The findings of the study suggest a possible association between age acceleration and ischemic stroke, as DNAm Age was observed to surpass chronological age in the cohort of individuals with ischemic stroke. The dissimilarity between methylation age and chronological age was most evident in individuals with cardioembolic stroke, with a median difference of 9 years. Conversely, the difference was least pronounced in those with atherothrombotic stroke, as depicted in Figure 1A–1D. Additionally, Andrew E. Teschendorff [34] and Jamaji C. Nwanaji-Enwerem [35], et al. used epiTOC2 to estimate the intrinsic stem cell proliferation rates in various normal tissue types, (Pearson correlation = 0.92, R² = 0.85, *P* = 3e−6), validating the association between methylation and the epigenetic mitotic clock.

### Divergent age acceleration among stroke subtypes

The analysis of stroke subtypes has revealed significant diversity in age acceleration. The study findings indicate that patients diagnosed with cardioembolic stroke demonstrated the most significant age acceleration, while those with small-vessel and atherothrombotic stroke exhibited a lower degree of age acceleration (as illustrated in Figure 1E–1H).

### Sex disparities in age acceleration

**Figure 2:**
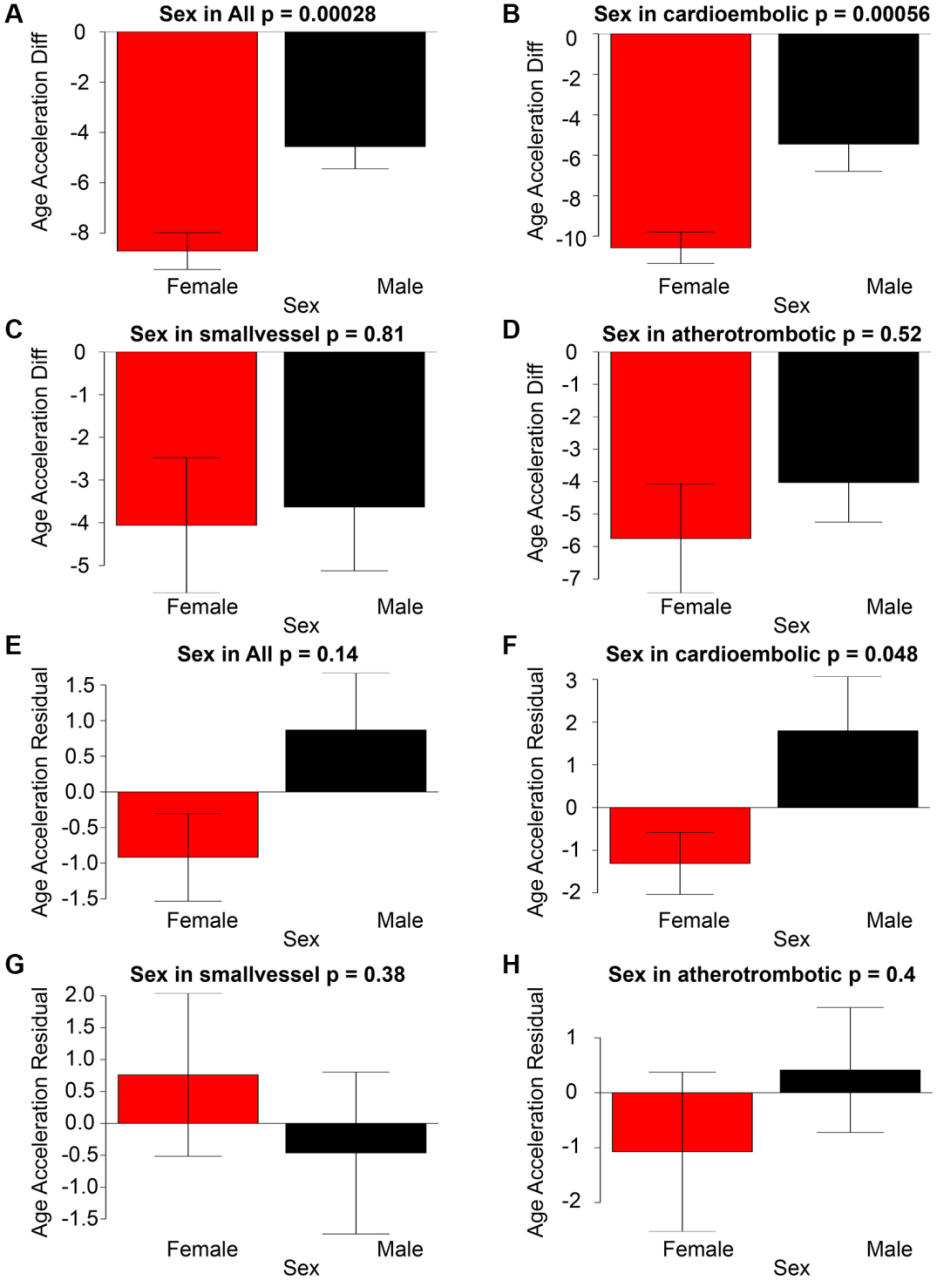
The graphical representation of Age Acceleration Diff (y-axis) in correlation with Sex for All stroke, cardioembolic stroke, small-vessel stroke, and atherothrombotic stroke can be observed in panels (**A**–**D**). Panels (**E**–**H**) illustrate the associations between Age Acceleration Residual (depicted on the y-axis) and Sex across All stroke, cardioembolic stroke, small-vessel stroke, and atherothrombotic stroke.

In general, there was a statistically significant difference in age acceleration between females and males. The observed disparity in gender was predominantly ascribed to individuals who had suffered from cardioembolic stroke. There were no statistically significant variations between sexes in residual-based age accelerations, as illustrated in Figure 2A–2H.

### The causal effect of stroke on age

**Figure 3:**
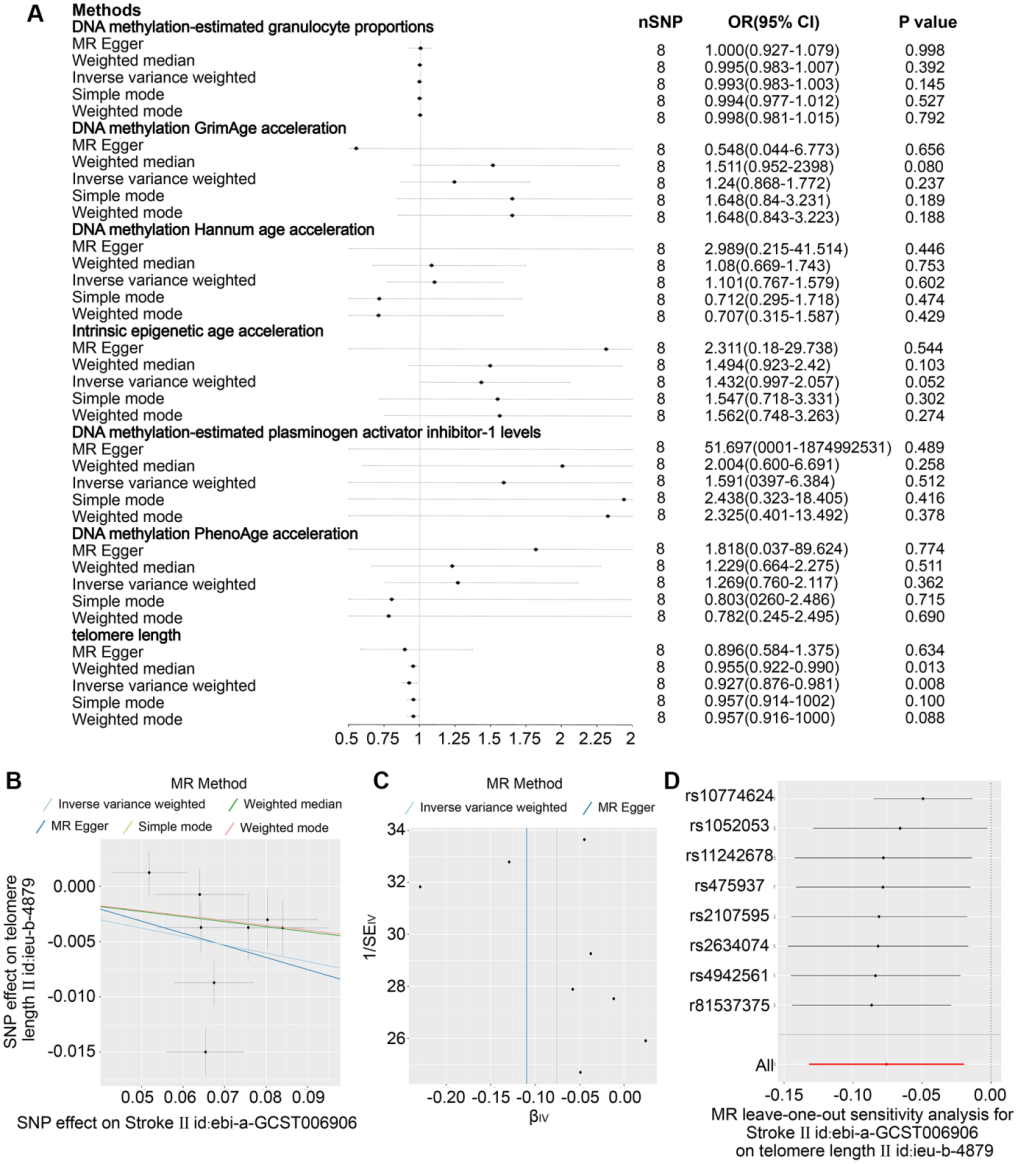
**The impact projections 6 DNA epigenetic modification-estimated traits (DNA epigenetic modification-estimated granulation cells dimensions, methylation of DNA GrimAge speed, DNA the methylation process Hannum age speed, Fundamental epigenetic modification age acceleration, which is DNA epigenetic regulation-estimated plasminogen stimulating activator inhibitor-1 levels, and DNA the methylation process PhenoAge acceleration) in stroke.** (**A**) Examination of the relationship between an increase in exposure to Stroke and the risk of DNA epigenetic regulation-estimated characteristics and Telomere length utilising Inverse variance weighed, MR Egger, Simple mode, Weighted mode and Weighted median estimates. (**B**) A scatter plot showing distinct SNP effects and predictions from various MR techniques regarding the impact of Stroke on Telomere duration. (**C**) Funnel illustrations of Telomere length and Stroke. (**D**) Leave-one-out research graphs for the effect of Stroke on Telomere duration.

**Figure 4:**
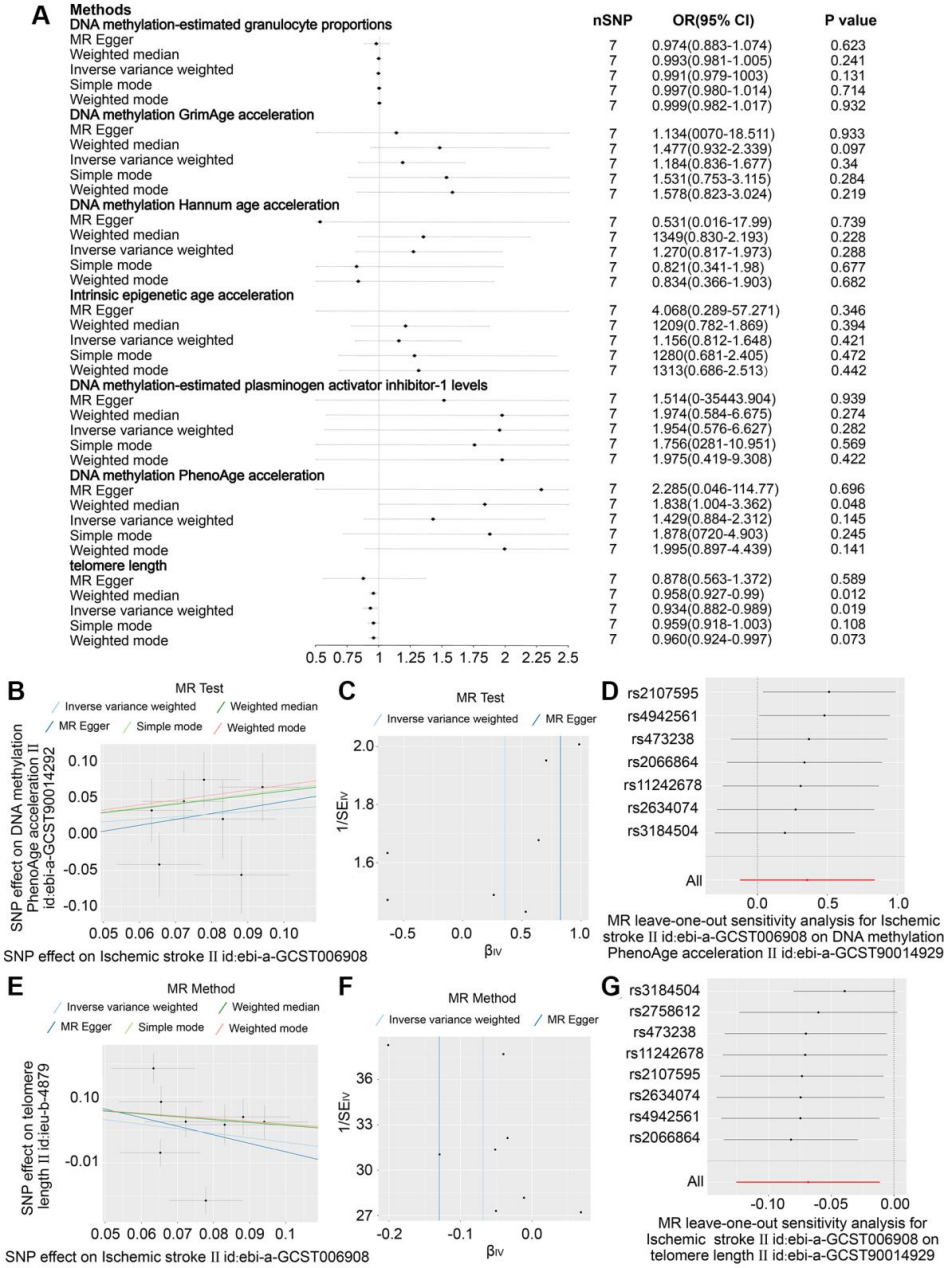
**In ischemic stroke, the effect estimates 6 DNA methylation-based phenotypes and telomere length.** (**A**) Examination of the association between an increase in ischemic stroke exposure and the risk of DNA histone modifications-estimated phenotypes and Telomere length, utilising Inverse variance weighed, MR Egger, Simple mode, Weighted mode and Weighted median estimates. (**B**) Scatter plots showing distinct SNP effects and approximations from various MR methods to investigate the effect of stroke caused by ischemic stroke on the acceleration of DNA methylation PhenoAge. (**C**) funnel Diagram of Ischemic Stroke on accelerated DNA methylation PhenoAge. (**D**) Leave-one-out regression diagrams for Ischemic Stroke and accelerated DNA methylation PhenoAge. (**E**) Scatter plots illustrating distinct SNP effects and estimates from various MR techniques depicting the influence of Ischemic Stroke on Telomere duration. (**F**) Ischemic Stroke funnel plots on Telomere length. (**G**) Leave-one-out evaluation plots for the effect of Ischemic Stroke on Telomere length.

Eight SNPs related to stroke were identified as robust genetic instruments with high confidence. The results presented in Figure 3A–3D demonstrate that the IVW revealed a negative causal relationship between stroke and telomere length (OR = 0.927 (95% CI, 0.876–0.981), *P* = 0.08). These findings suggest that both stroke and ischemic stroke may have a detrimental effect on telomere length. The study yielded comparable findings for ischemic stroke (OR = 0.934 (95% CI, 0.882–0.989), *P* = 0.019) as depicted in Figure 4A, 4E–4G. Nonetheless, no causal relationship was established between LAS, CES, and SVS and telomere length, as illustrated in Supplementary Figures 1–8. The sole statistical method that demonstrated a favorable causal impact of ischemic stroke on PhenoAge was the Weighted Median (OR = 1.838 (95% CI, 1.004–03.362), *P* = 0.048) (Figure 4B–4D). This suggests that ischemic stroke may potentially hasten the process of aging as measured by PhenoAge. No significant causal relationship was observed between stroke and its subtypes with intrinsic epigenetic age acceleration, including HannumAge, PhenoAge, GrimAge, and PAI1.

### The causal effect of age on stroke

A reverse Mendelian randomization analysis was conducted to provide additional elucidation on the causal impact of age on stroke, with age serving as the exposure and stroke as the outcome. The study’s findings indicate that there is no discernible causal relationship between age phenotypes and the incidence of stroke, ischemic stroke, LAS, CES, and SVS.

## DISCUSSION

Epigenetic age acceleration, which refers to the variance between DNAm Age and chronological age, serves as a surrogate for biological aging and has been associated with various age-related illnesses [49]. Furthermore, it has been shown that methylation markers exhibit considerable potential in forecasting telomere length, which serves as a molecular indicator of cellular aging [50, 51]. The correlation between epigenetic age acceleration and the risk of ischemic stroke, with regards to stroke subtypes and gender-specific variations, has not been extensively investigated, despite the emergence of supporting evidence [15, 16].

The present study furnishes empirical support for the correlation between epigenetic age acceleration and ischemic stroke, with the most notable disparities being discerned in individuals afflicted with cardioembolic stroke. Cardioembolic strokes are typically caused by heart diseases such as atrial fibrillation, which can lead to systemic inflammatory responses. Systemic inflammation can accelerate biological aging by affecting DNA methylation and other epigenetic mechanisms through various pathways. Inflammatory markers such as C-reactive protein (CRP) and interleukin-6 (IL-6) have been shown to be associated with accelerated epigenetic aging [29, 52]. Cardioembolic strokes typically involve thrombus formation in the systemic blood circulation, suggesting that their impact may not be limited to the cerebral vascular system but also involve epigenetic changes in multiple organs and systems throughout the body [28, 53]. This systemic impact may manifest epigenetic aging acceleration more prominently than localized vascular lesions. The pathological mechanisms of large artery atherosclerotic stroke and small vessel disease stroke differ from those of cardioembolic stroke. Large artery atherosclerotic stroke is mainly due to plaque formation and rupture in large vessels, whereas small vessel disease stroke is due to pathology in cerebral small arteries. Although these pathological processes also affect epigenetics, their effects may differ in manner and degree from cardioembolic stroke [54].

In addition, our study revealed the presence of gender-based differences in age acceleration, with females displaying a significantly greater degree of age acceleration in comparison to males. This discrepancy was mainly attributed to cardioembolic stroke. The Mendelian randomization analysis conducted in our study has indicated a negative causal impact of stroke on telomere length. This finding suggests that stroke may be a contributing factor to the reduction of telomere length. Nevertheless, the study did not identify any causal relationships between age phenotypes and the risk of stroke. The correlation between methylation age acceleration and ischemic stroke, with a specific focus on female patients and those with cardioembolic stroke, underscores the significance of incorporating biological age into stroke risk factor research, alongside chronological age. Further investigations are necessary to comprehend the underlying mechanisms that contribute to sexual dimorphism in stroke risk and outcomes, specifically regarding sex-specific differences in age acceleration.

The objective of our study was to employ a two-sample bidirectional Mendelian randomization approach to establish the causal association between different measures of epigenetic age acceleration and stroke, along with its subtypes. In general, our findings do not provide robust evidence to establish a causal relationship between age acceleration and stroke, or the reverse. Nonetheless, it was noted that there may be a plausible causal relationship between ischemic stroke and the acceleration of PhenoAge. This finding necessitates additional research to be conducted. The paucity of evidence substantiating a causal association between epigenetic age acceleration and stroke could be ascribed to various factors. The potential insufficiency of sample sizes in the available GWAS data may have hindered the detection of weak causal effects. Furthermore, the study’s methodology may have potentially introduced survivorship bias, given that solely those individuals who survived until the age of stroke ascertainment were incorporated. In addition, despite the implementation of various MR techniques to ensure the reliability of the analysis and to address the possibility of pleiotropy, it is plausible that our findings may have been affected by unmeasured or residual confounding. Finally, it should be noted that our analysis was centered on stroke and its prevalent subtypes, and therefore, it is not possible to completely eliminate the likelihood of causal associations within particular, more limited stroke subcategories that were not investigated in this research.

The potential causal effect of ischemic stroke on PhenoAge acceleration, as found by our study, warrants further attention. The PhenoAge clock has demonstrated a stronger correlation with disease and mortality risk in comparison to other epigenetic clocks, as evidenced by previous studies [55]. The observed correlation could potentially be attributed to biomarkers that are linked to both stroke and expedited aging. Individuals with accelerated PhenoAge have been shown to exhibit higher levels of inflammation, which is a recognized risk factor for stroke [56, 57]. Additional investigation is warranted to examine the common biological mechanisms that underlie both stroke and accelerated aging. DNA methylation is associated with the regulation of cell apoptosis and repair mechanisms. Accelerated DNA methylation age may lead to increased cell apoptosis and reduced repair capacity, thereby increasing the risk of stroke [6]. Additionally, it is important to explore the potential ameliorative impact of lifestyle modifications and pharmacological interventions on this association.

The main findings of this study revealed a significant association between DNA methylation estimated phenotype and telomere length with the risk of ischemic stroke. Identifying DNA methylation sites and telomere length associated with the risk of ischemic stroke facilitates the development of new screening tools. These epigenetic markers can be used for early detection of high-risk individuals, thereby implementing preventive measures. For instance, combining Horvath’s methylation clock model with telomere length measurements could introduce epigenetics-based screening methods into routine medical examinations [6]. This helps identify individuals who have not yet shown obvious symptoms but have a higher risk of stroke. Based on the screening results of epigenetic markers, personalized prevention strategies can be developed. For instance, for individuals at high risk due to accelerated DNA methylation age and shortened telomeres, more frequent health monitoring and early interventions such as blood pressure control, lipid-lowering medication, and lifestyle changes can be recommended [58].

The principal advantage of our investigation is the implementation of a two-sample MR analysis, which permits the examination of reciprocal causal associations while mitigating the potential confounding influences frequently encountered in observational research. Nonetheless, our research encountered certain constraints. As previously stated, it is possible that the sample sizes and outcome measures utilized in the study were not adequately detailed or inclusive enough to account for more subtle causal impacts. Furthermore, the limited representation of non-European samples in the subject matter may restrict the generalizability of our results. Our two-sample MR analysis yielded insufficient evidence to establish a causal relationship between methylation age acceleration and stroke, indicating the need for further inquiry utilizing larger sample sizes and more precise biological age assessments. Enhancing our understanding of stroke pathophysiology necessitates a comprehensive comprehension of the interplay between chromosomal age acceleration and stroke risk, which may pave the way for the emergence of innovative stroke prevention and treatment modalities.

## CONCLUSION

The study’s results suggest that Epigenetic age acceleration may play a role in the risk of ischemic stroke, particularly in relation to subtype-specific associations and gender disparities. The findings of this study suggest the need for further investigation into the biological mechanisms that underlie the associations between age acceleration, stroke subtypes, and sex. This could potentially lead to the use of methylation-based age biomarkers in stroke risk stratification and prevention strategies.

## Supplementary Materials

Supplementary Figures
