# Supplementary material for: DNA methylation-estimated phenotypes, telomere length and risk of ischemic stroke: epigenetic age acceleration of screening and a Mendelian randomization study
Source: Aging (Albany NY). 2024 Aug 16;16(16):11970–93. doi: 10.18632/aging.206072 (PMC11386914; doi:10.18632/aging.206072)
Supplement: Supplementary Figures [file aging-16-206072-s001.pdf]

SUPPLEMENTARY FIGURES

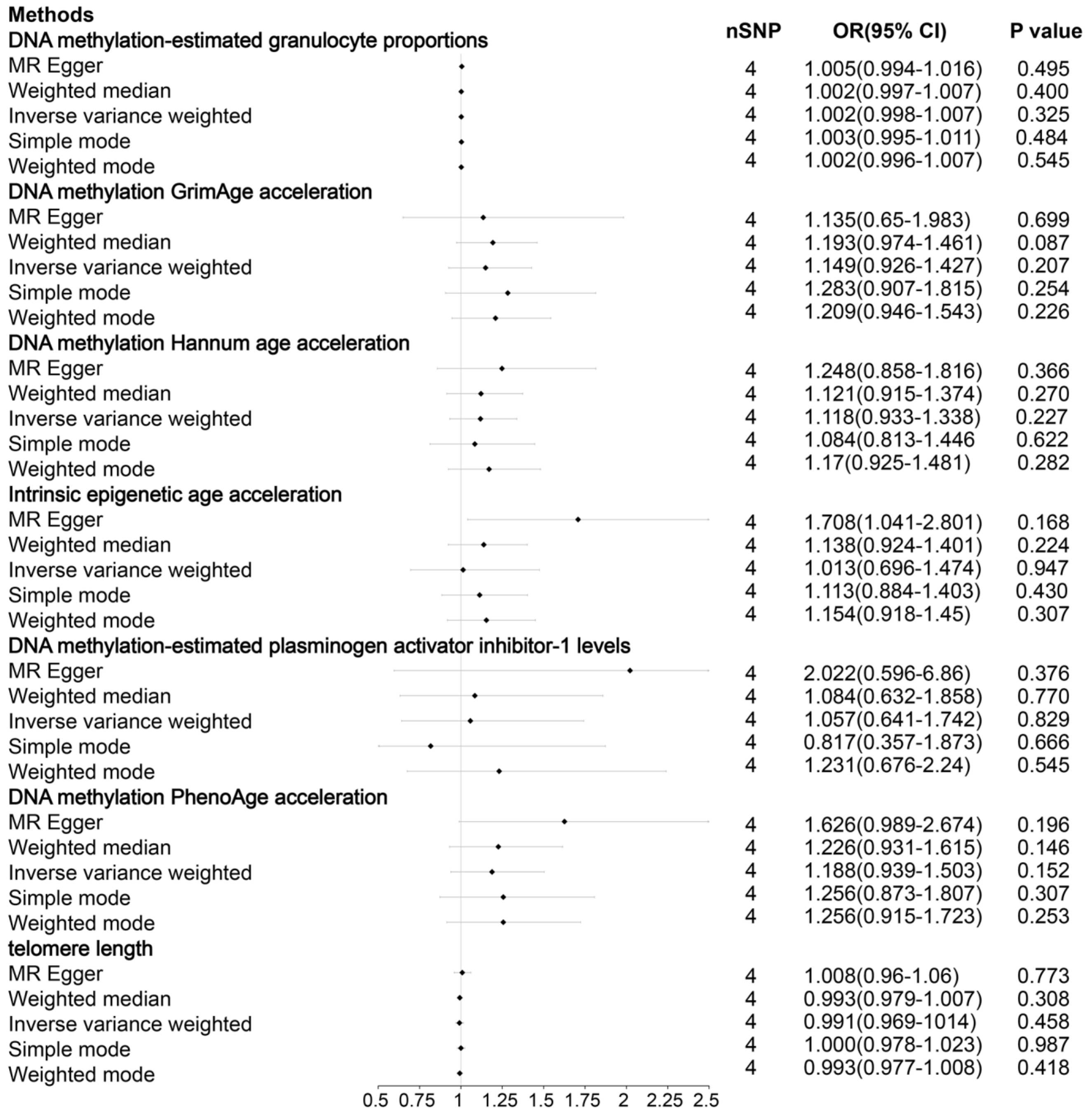

Supplementary Figure 1. Examination of the association between an increase in Cardioembolic stroke exposure and the risk of DNA histone modifications-estimated phenotypes and Telomere length, utilising Inverse variance weighed, MR Egger, Simple mode, Weighted mode and Weighted median estimates.

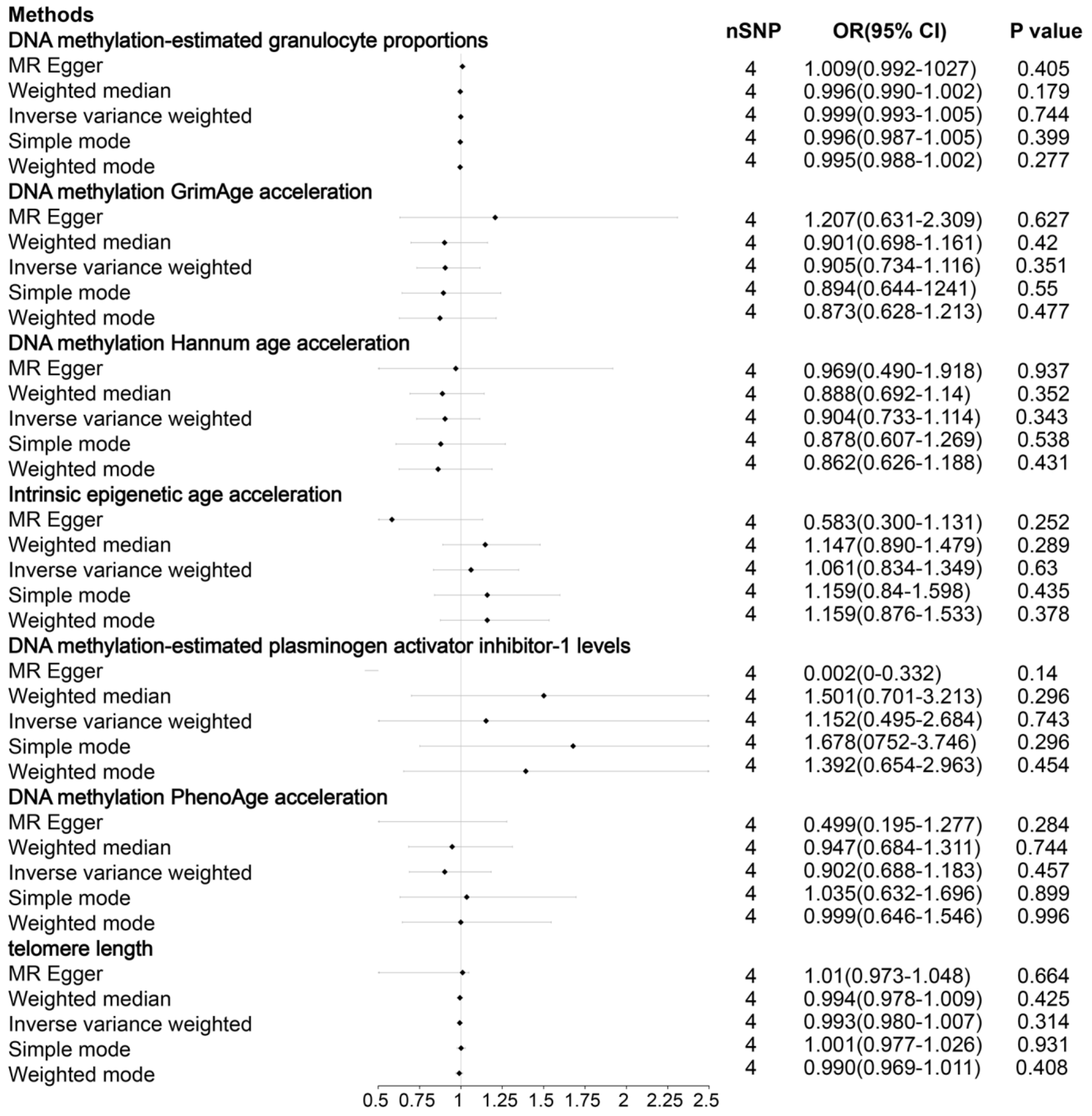

Supplementary Figure 2. Examination of the association between an increase in Large-artery atherosclerosis stroke exposure and the risk of DNA histone modifications-estimated phenotypes and Telomere length, utilising Inverse variance weighed, MR Egger, Simple mode, Weighted mode and Weighted median estimates.

Methods	nSNP	OR(95% CI)	P value
**DNA methylation-estimated granulocyte proportions**			
MR Egger	31	1.001(0.996-1.006)	0.699
Weighted median	31	1.003(1.000-1.006)	0.067
Inverse variance weighted	31	1.002(1.000-1.004)	0.078
Simple mode	31	1.004(0.998-1.011)	0.215
Weighted mode	31	1.004(0.998-1.010)	0.188
**DNA methylation GrimAge acceleration**			
MR Egger	31	0.798(0.630-1.012)	0.072
Weighted median	31	0.934(0.810-1.075)	0.341
Inverse variance weighted	31	0.941(0.846-1.047)	0.267
Simple mode	31	0.937(0.710-1.237)	0.650
Weighted mode	31	0.946(0.727-1.230)	0.681
**DNA methylation Hannum age acceleration**			
MR Egger	31	1.296(1.026-1.637)	0.038
Weighted median	31	1.017(0.893-1.158)	0.798
Inverse variance weighted	31	0.988(0.882-1.107)	0.841
Simple mode	31	1.029(0.817-1.296)	0.810
Weighted mode	31	1.058(0.829-1.351)	0.651
**Intrinsic epigenetic age acceleration**			
MR Egger	31	1.207(0.968-1.505)	0.106
Weighted median	31	0.986(0.857-1.134)	0.841
Inverse variance weighted	31	0.968(0.877-1.068)	0.514
Simple mode	31	1.008(0.776-1.309)	0.954
Weighted mode	31	1.000(0.760-1.315)	0.999
**DNA methylation-estimated plasminogen activator inhibitor-1 levels**			
MR Egger	31	0.896(0.267-3.007)	0.860
Weighted median	31	1.299(0.870-1.940)	0.201
Inverse variance weighted	31	1.319(0.967-1.798)	0.081
Simple mode	31	0.909(0.248-3.334)	0.886
Weighted mode	31	0.909(0.243-3.401)	0.888
**DNA methylation PhenoAge acceleration**			
MR Egger	31	0.986(0.721-1.348)	0.932
Weighted median	31	0.947(0.791-1.133)	0.549
Inverse variance weighted	31	0.967(0.845-1.105)	0.620
Simple mode	31	0.988(0.700-1.395)	0.945
Weighted mode	31	1.058(0760-1.474)	0.741
**telomere length**			
MR Egger	31	1.003(0.989-1018)	0.648
Weighted median	31	1.003(0.994-1.011)	0.531
Inverse variance weighted	31	1.006(0.999-1.014)	0.079
Simple mode	31	1.003(0.984-1.021)	0.785
Weighted mode	31	1.001(0.982-1.020)	0.924

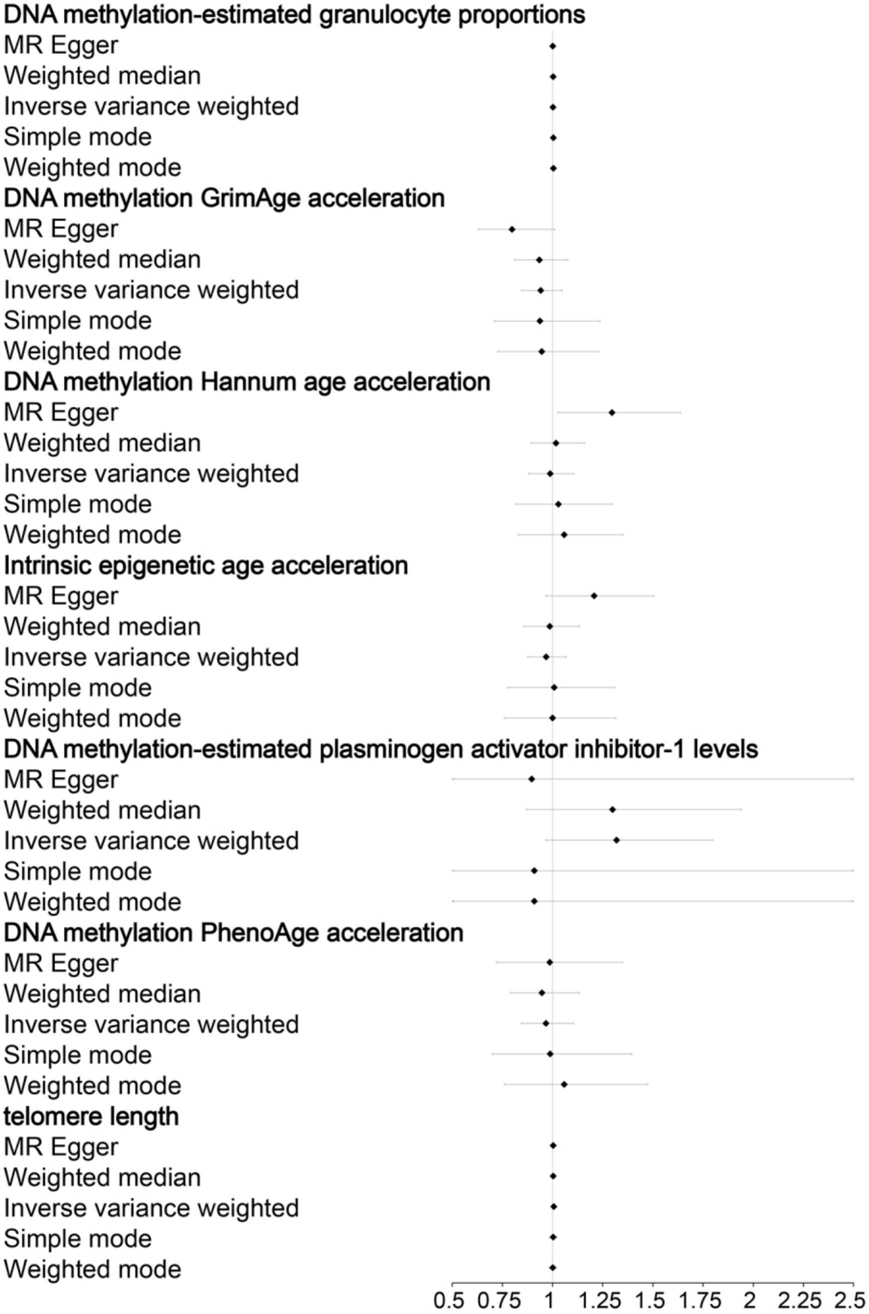

Supplementary Figure 3. Examination of the association between an increase in Small-vessel stroke exposure and the risk of DNA histone modifications-estimated phenotypes and Telomere length, utilising Inverse variance weighed, MR Egger, Simple mode, Weighted mode and Weighted median estimates.

Methods	nSNP	OR(95% CI)	P value
**DNA methylation-estimated granulocyte proportions**			
Inverse variance weighted	2	0.301(0-50089.867)	0.845
**DNA methylation GrimAge acceleration**			
MR Egger	4	2.227(0.243-20.377)	0.552
Weighted median	4	0.959(0.832-1.106	0.564
Inverse variance weighted	4	0.945(0.831-1.075)	0.391
Simple mode	4	0.902(0.72-1.132)	0.439
Weighted mode	4	1003(0.829-1213)	0.977
**DNA methylation Hannum age acceleration**			
MR Egger	8	0.769(0.604-0.979)	0.077
Weighted median	8	0.947(0.869-1.032)	0.217
Inverse variance weighted	8	0.947(0.886-1011)	0.103
Simple mode	8	0.93(0.807-1.072)	0.348
Weighted mode	8	0.925(0.823-1.039)	0.232
**Intrinsic epigenetic age acceleration**			
MR Egger	24	0.996(0.889-1.116)	0.943
Weighted median	24	101(0.959-1.064)	0.715
Inverse variance weighted	24	1028(0.987-1.071)	0.188
Simple mode	24	1.055(0.935-1.19)	0.395
Weighted mode	24	0.993(0.91-1084)	0.883
**DNA methylation-estimated plasminogen activator inhibitor-1 levels**			
MR Egger	5	1(1-1)	0.705
Weighted median	5	1(1-1)	0.321
Inverse variance weighted	5	1(1-1)	0.858
Simple mode	5	1(1-1)	0.371
Weighted mode	5	1(1-1)	0.409
**DNA methylation PhenoAge acceleration**			
MR Egger	11	0.957(0.869-1.054)	0.393
Weighted median	11	0.977(0.926-1.031)	0.403
Inverse variance weighted	11	0.996(0.956-1037)	0.839
Simple mode	11	0.943(0.849-1047)	0.300
Weighted mode	11	0.952(0.879-1032)	0.259
**telomere length**			
MR Egger	133	1.01(0.73-1.398)	0.954
Weighted median	133	1.011(0.773-1322)	0.938
Inverse variance weighted	133	1.015(0.849-1.214)	0.867
Simple mode	133	0.829(0.44-1.561)	0.562
Weighted mode	133	0.977(0.655-1.457)	0.909

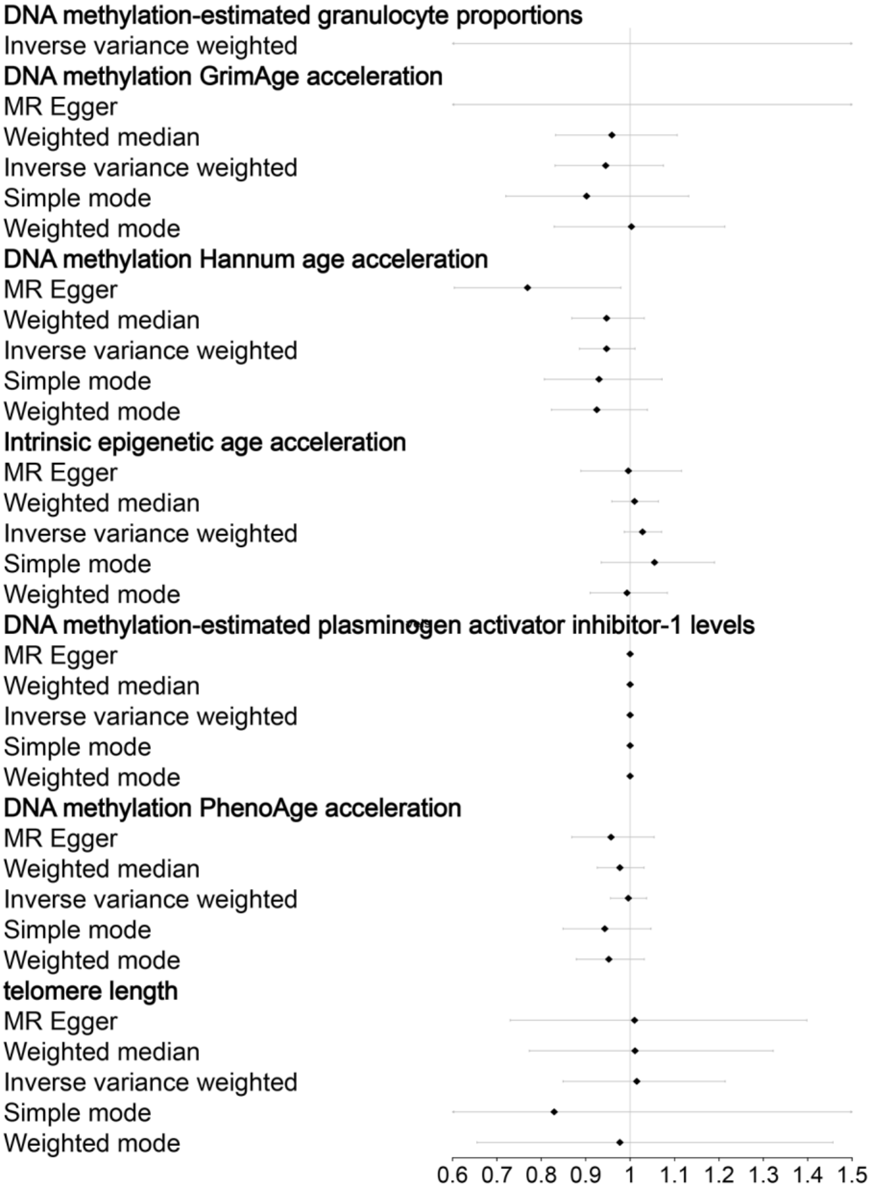

Supplementary Figure 4. Analysis of the association between DNA methylation-estimated phenotypes and Telomere length exposure and the risk of Cardioembolic stroke using Inverse variance weighed, MR Egger, Simple mode, Weighted mode and Weighted median estimates.

Methods	nSNP	OR(95% CI)	P value
**DNA methylation-estimated granulocyte proportions**			
Inverse variance weighted	2	0.091(0-71.62)	0.481
**DNA methylation GrimAge acceleration**			
MR Egger	4	0.184(0.012-2.922)	0.353
Weighted median	4	0.909(0.753-1.097	0.319
Inverse variance weighted	4	0.839(0702-1.001)	0.052
Simple mode	4	0.928(0.701-1.228)	0.637
Weighted mode	4	0.9210.699-1.214)	0.600
**DNA methylation Hannum age acceleration**			
MR Egger	8	0.752(0.496-1.139)	0.227
Weighted median	8	1.032(0.925-1.151)	0.576
Inverse variance weighted	8	1.043(0.922-1.181)	0.503
Simple mode	8	1.032(0.89-1.198)	0.687
Weighted mode	8	1.025(0.91-1.155)	0.692
**Intrinsic epigenetic age acceleration**			
MR Egger	24	1.1(0.971-1.246)	0.148
Weighted median	24	1013(0.948-1.082)	0.700
Inverse variance weighted	24	1.012(0.966-1.06)	0.628
Simple mode	24	0.937(0.828-1.062)	0.319
Weighted mode	24	0.935(0.838-1.045)	0.252
**DNA methylation-estimated plasminogen activator inhibitor-1 levels**			
MR Egger	5	1(1-1)	0.718
Weighted median	5	1(1-1)	0.792
Inverse variance weighted	5	1(1-1)	0.669
Simple mode	5	1(1-1)	0.529
Weighted mode	5	1(1-1)	0.917
**DNA methylation PhenoAge acceleration**			
MR Egger	11	0.953(0.793-1.144)	0.616
Weighted median	11	0.936(0.87-1.006)	0.073
Inverse variance weighted	11	0.961(0.895-1.032)	0.277
Simple mode	11	0.904(0.8-1.02)	0.133
Weighted mode	11	0.908(0.816-1.01)	0.107
**telomere length**			
MR Egger	133	0.915(0.6-1398)	0.683
Weighted median	133	0.849(0.615-1.173)	0.321
Inverse variance weighted	133	0.912(0723-1151)	0.437
Simple mode	133	1.004(0.522-1.931)	0.991
Weighted mode	133	1.004(0.686-1.468)	0.985

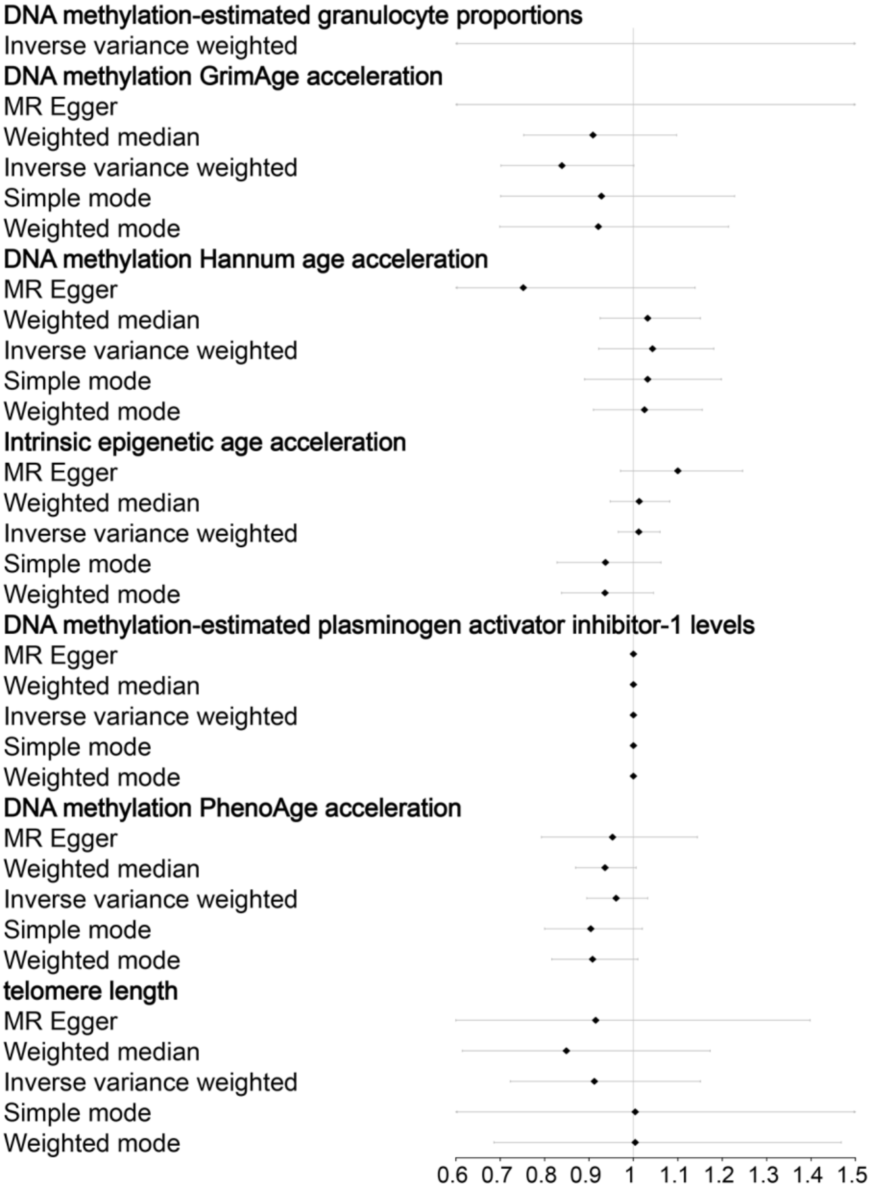

Supplementary Figure 5. Analysis of the association between DNA methylation-estimated phenotypes and Telomere length exposure and the risk of Large-artery atherosclerosis stroke using Inverse variance weighed, MR Egger, Simple mode, Weighted mode and Weighted median estimates.

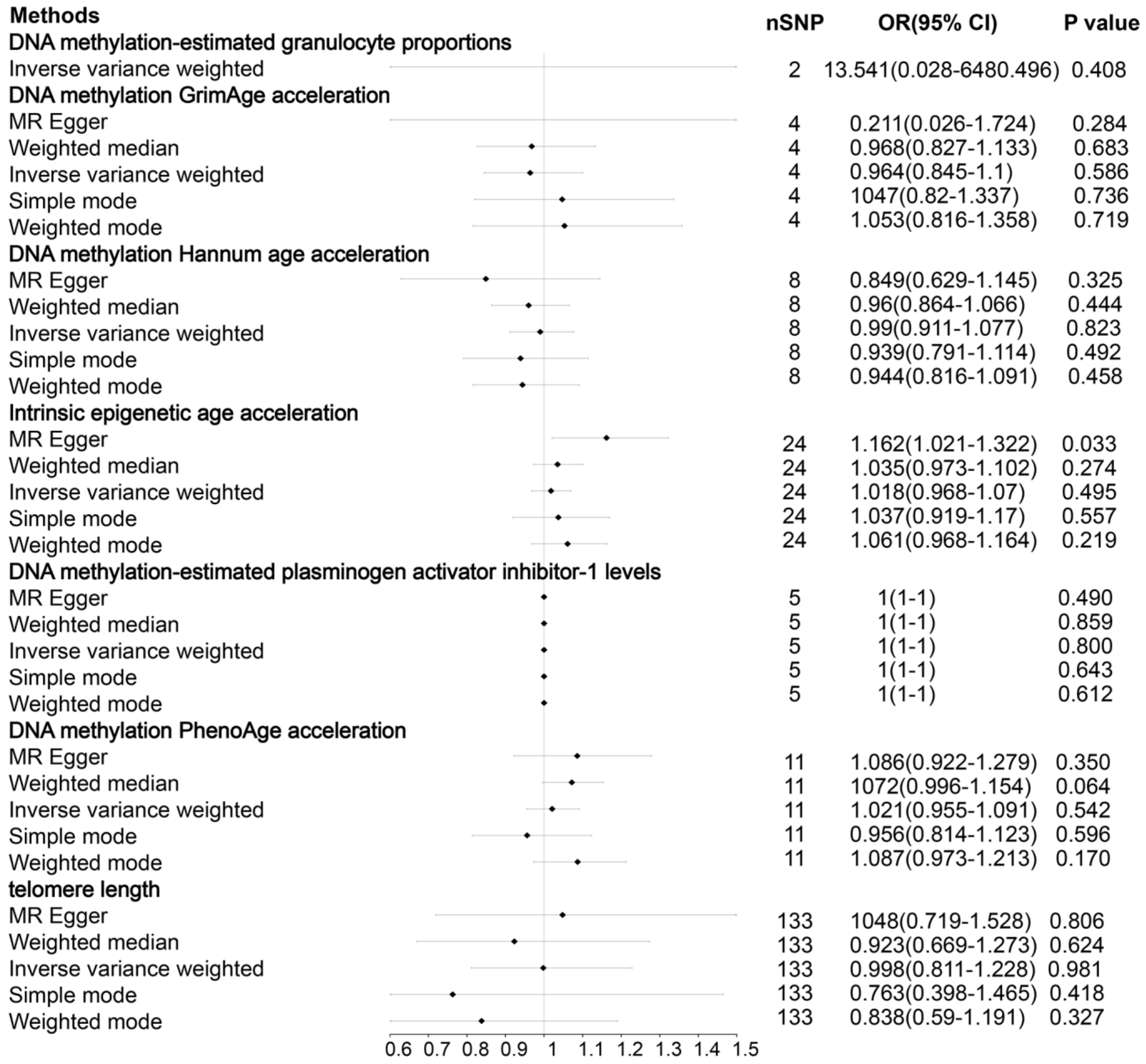

Supplementary Figure 6. Analysis of the association between DNA methylation-estimated phenotypes and Telomere length exposure and the risk of Small-vessel stroke using Inverse variance weighed, MR Egger, Simple mode, Weighted mode and Weighted median estimates.

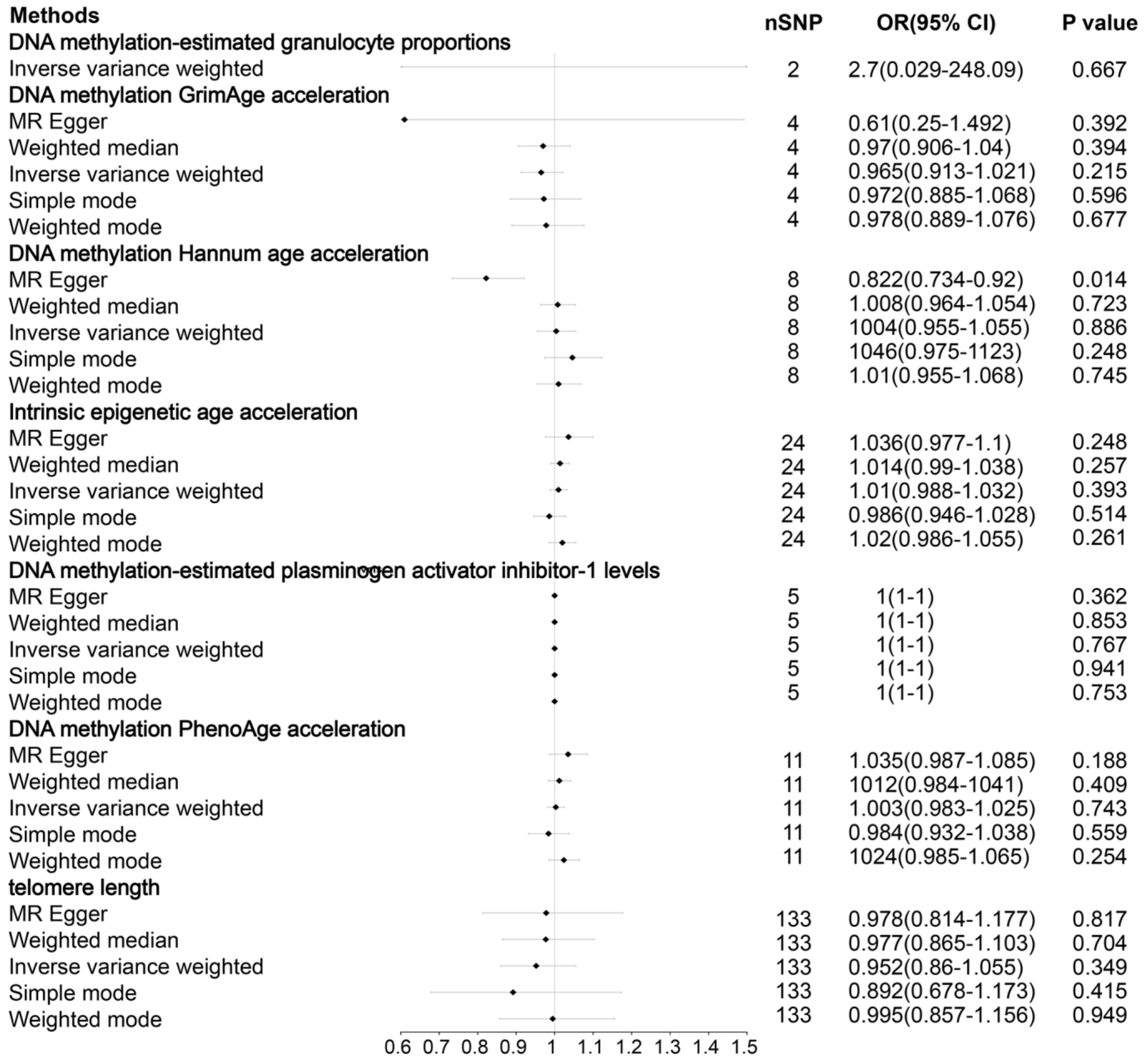

Supplementary Figure 7. Analysis of the association between DNA methylation-estimated phenotypes and Telomere length exposure and the risk of Stroke using Inverse variance weighed, MR Egger, Simple mode, Weighted mode and Weighted median estimates.

Methods	nSNP	OR(95% CI)	P value
**DNA methylation-estimated granulocyte proportions**			
Inverse variance weighted	2	4.684(0.066-332.838)	0.478
**DNA methylation GrimAge acceleration**			
MR Egger	4	0.587(0.233-1.475)	0.375
Weighted median	4	0.977(0.911-1.048)	0.518
Inverse variance weighted	4	0.983(0.928-1.042)	0.565
Simple mode	4	0.967(0.867-1.079)	0.593
Weighted mode	4	0.958(0 868-1.058)	0.458
**DNA methylation Hannum age acceleration**			
MR Egger	8	0.825(0.73-0.933)	0.022
Weighted median	8	1.002(0.957-1.048)	0.945
Inverse variance weighted	8	0.997(0.947-1.049)	0.913
Simple mode	8	1.037(0.973-1.105)	0.305
Weighted mode	8	1.001(0.949-1.056)	0.971
**Intrinsic epigenetic age acceleration**			
MR Egger	24	1.041(0.975-1.112)	0.242
Weighted median	24	1.017(0.99-1.045)	0.216
Inverse variance weighted	24	1.005(0.981-1.03)	0.68
Simple mode	24	0.982(0.936-1.029)	0.449
Weighted mode	24	1.029(0.992-1.068)	0.137
**DNA methylation-estimated plasminogen activator inhibitor-1 levels**			
MR Egger	5	1(1-1)	0.298
Weighted median	5	1(1-1)	0.764
Inverse variance weighted	5	1(1-1)	0.851
Simple mode	5	1(1-1)	0.746
Weighted mode	5	1(1-1)	0.375
**DNA methylation PhenoAge acceleration**			
MR Egger	11	1.039(0.989-1.091)	0.162
Weighted median	11	1.008(0.978-1039)	0.622
Inverse variance weighted	11	1.004(0.983-1.026)	0.689
Simple mode	11	1(0.95-1.054)	0.989
Weighted mode	11	1.027(0.983-1.074)	0.258
**telomere length**			
MR Egger	133	0.983(0.811-1.193)	0.865
Weighted median	133	0.988(0.864-1.129)	0.857
Inverse variance weighted	133	0.99(0.89-1.101)	0.851
Simple mode	133	0.961(0 729-1.268)	0.781
Weighted mode	133	0.997(0.859-1.157)	0.966

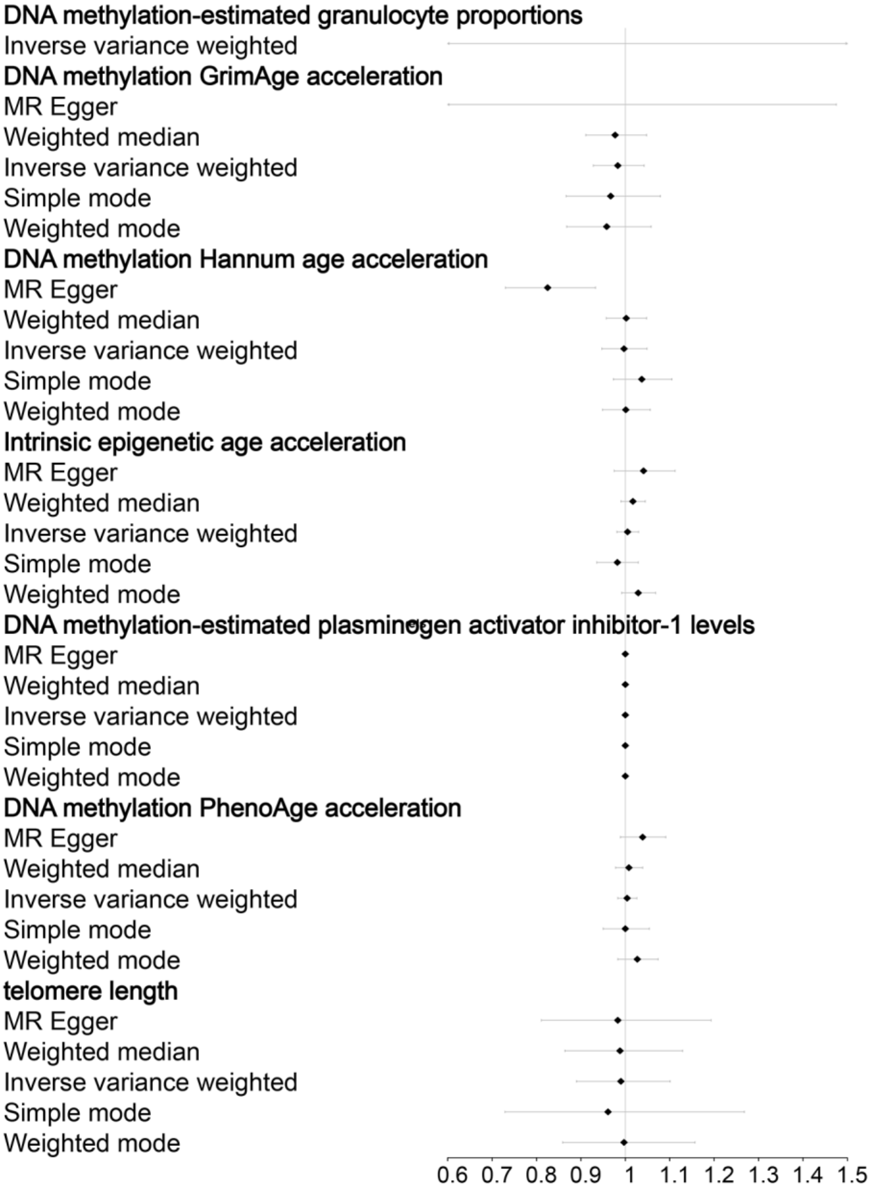

Supplementary Figure 8. Analysis of the association between DNA methylation-estimated phenotypes and Telomere length exposure and the risk of Ischemic stroke using Inverse variance weighed, MR Egger, Simple mode, Weighted mode and Weighted median estimates.
